# Reducing the socioeconomic gradient in uptake of the NHS bowel cancer screening Programme using a simplified supplementary information leaflet: a cluster-randomised trial

**DOI:** 10.1186/s12885-017-3512-1

**Published:** 2017-08-14

**Authors:** Samuel G. Smith, Jane Wardle, Wendy Atkin, Rosalind Raine, Lesley M. McGregor, Gemma Vart, Steve Morris, Stephen W. Duffy, Susan Moss, Allan Hackshaw, Stephen Halloran, Ines Kralj-Hans, Rosemary Howe, Julia Snowball, Graham Handley, Richard F. Logan, Sandra Rainbow, Steve Smith, Mary Thomas, Nicholas Counsell, Christian von Wagner

**Affiliations:** 1 Leeds Institute of Health Sciences, University of Leeds, Leeds, LS2 9LB UK; 2 Department of Behavioural Science and Health, University College London, London, WC1E 7HB UK; 3 Faculty of Medicine, Department of Surgery & Cancer, Imperial College London, London, W2 1NY UK; 4 Department of Applied Health Research, University College London, London, WC1E 7HB UK; 5 Research & Enterprise Royal Holloway University of London, London, England; 6 Wolfson Institute of Preventive Medicine, Queen Mary University of London, London, EC1M 6BQ UK; 7 Cancer Research UK & UCL Cancer Trials Centre, University College London, London, WC1E 7HB UK; 8 Bowel Cancer Screening Southern Programme Hub, Guildford, GU2 7XX UK; 9 Academic Neuroscience Centre, King’s College London, London, SE5 8AF UK; 10 North East Bowel Cancer Screening Hub, Gateshead, NE9 6SX UK; 11 Eastern Hub of the Bowel Cancer Screening Programme, Nottingham, NG7 2UH UK; 12 Bowel Cancer Screening Programme London Programme Hub, London, HA1 3UJ UK; 13 Midlands & North West Bowel Cancer Screening Programme Hub, Rugby, CV22 5PX UK

**Keywords:** Cancer; oncology; socioeconomic inequalities, Colorectal cancer screening, Fuzzy trace theory, Gist

## Abstract

**Background:**

Uptake of colorectal cancer screening is low in the English NHS Bowel Cancer Screening Programme (BCSP). Participation in screening is strongly associated with socioeconomic status. The aim of this study was to determine whether a supplementary leaflet providing the ‘gist’ of guaiac-based Faecal Occult Blood test (gFOBt) screening for colorectal cancer could reduce the socioeconomic status (SES) gradient in uptake in the English NHS BCSP.

**Methods:**

The trial was integrated within routine BCSP operations in November 2012. Using a cluster randomised controlled design all adults aged 59–74 years who were being routinely invited to complete the gFOBt were randomised based on day of invitation. The Index of Multiple Deprivation was used to create SES quintiles. The control group received the standard information booklet (‘SI’). The intervention group received the SI booklet and the Gist leaflet (‘SI + Gist’) which had been designed to help people with lower literacy engage with the invitation. Blinding of hubs was not possible and invited subjects were not made aware of a comparator condition. The primary outcome was the gradient in uptake across IMD quintiles.

**Results:**

In November 2012, 163,525 individuals were allocated to either the ‘SI’ intervention (*n* = 79,104) or the ‘SI + Gist’ group (*n* = 84,421). Overall uptake was similar between the intervention and control groups (SI: 57.3% and SI + Gist: 57.6%; OR = 1.02, 95% CI: 0.92–1.13, *p* = 0.77). Uptake was 42.0% (SI) vs. 43.0% (SI + Gist) in the most deprived quintile and 65.6% vs. 65.8% in the least deprived quintile (interaction *p* = 0.48). The SES gradient in uptake was similar between the study groups within age, gender, hub and screening round sub-groups.

**Conclusions:**

Providing supplementary simplified information in addition to the standard information booklet did not reduce the SES gradient in uptake in the NHS BCSP. The effectiveness of the Gist leaflet when used alone should be explored in future research.

**Trial registration:**

ISRCTN74121020, registered: 17/20/2012.

**Electronic supplementary material:**

The online version of this article (doi:10.1186/s12885-017-3512-1) contains supplementary material, which is available to authorized users.

## Background

Biennial screening using guaiac-based Faecal Occult Blood testing (gFOBT) reduces colorectal cancer (CRC) mortality [1]. The National Health Service (NHS) Bowel Cancer Screening Programme (BCSP) in England offers biennial CRC screening by gFOBt to all adults aged 60–74 years. Uptake from 2006 to 2009 was 54%, [2] which is lower than the breast (73%) and cervical programmes (79%) [3, 4]. These data also demonstrate a strong gradient in uptake by socioeconomic status (SES), with uptake ranging from 35% in the most deprived area quintile to 61% in the least deprived quintile [2]. Adherence to follow-up procedures is high (88%) and shows little association with SES [5].

Low engagement with screening information may partially explain disappointing uptake rates [6]. A large proportion of people in deprived areas have low literacy skills [7] and information materials may be too complex to facilitate informed decision-making [8–11]. Difficulties with comprehending the existing information booklet, ‘Bowel Cancer Screening: The Facts’ may explain why limited literacy is a risk factor for sub-optimal participation [12–14]. Multiple socioeconomic factors affect screening participation, however literacy has been shown to be an independent predictor of uptake after adjusting for age, sex, education, occupation, ethnicity and wealth [12]. Literacy-related barriers can be addressed face-to-face or by telephone contact, [15] but this is not practical within a national screening programme.

Psychological models argue that decision-making can be improved for people with poor literacy by providing the ‘gist’ of information (e.g. ‘screening saves lives’) [16]. Highlighting the ‘gist’ of screening and removing unnecessary information could improve the ease with which screening decisions can be reached, particularly for lower socioeconomic status groups. We developed a gist-based information leaflet that begins with statements encapsulating the main aims of CRC screening, followed by key information in simple language [17]. In line with NHS policy, the Gist leaflet was sent as a supplement to the standard information booklet. We hypothesised that the Gist leaflet would be progressively more effective in improving screening uptake with increasing levels of area-based socioeconomic deprivation.

## Methods

The study was a two-arm, cluster-randomised trial with individuals routinely invited for CRC screening within the NHS BCSP. They received either: the standard information booklet (SI); or, the standard booklet plus the supplementary Gist leaflet (SI + Gist). The trial had multicentre ethics approval from the National Research Ethics Service Committee London-Harrow (REC ref.: 12/LO/1396). The Cancer Screening Programmes are covered by National Information Governance Board (NIGB) approval for handling patient-identifiable data. The trial was prospectively registered on the 17th October, 2012 (ISRCTN74121020). We adhered to the Consort guidelines throughout.

### Setting and participants

The administration of the BCSP is co-ordinated by five regional centres or ‘hubs’. Each hub sends an invitation and the screening information every 2 years from the 60th birthday to all patients registered with a General Practitioner (GP) in their region. The gFOBt kit is sent 8–10 days later, along with instructions on how to perform the test. To participate in screening, the individual collects small samples from three bowel motions, and returns the kit to the hub in a pre-paid envelope. A reminder is sent after 4 weeks to those who have not responded. If there has not been a response to the invitation after 13 weeks, the ‘screening episode’ is closed. The hubs process the kits and the result is sent to the individual and their GP within 2 weeks. Routine gFOB testing is offered 2 years later for those with a normal result. A repeat test is sent for a spoilt kit, a technical fail, or an unclear result. Each hub works with up to 18 local screening centres which are responsible for providing follow-up investigations for individuals with abnormal results.

This trial involved all five hubs and included all individuals invited during the study period. Individuals not registered with a GP (~4% of the population) were not included, and those who opted out of screening were not sent further kits. People undergoing investigation for colorectal problems or who had undergone bowel surgery are requested to seek advice from a helpline.

### Intervention

*Control group: Standard Information booklet (SI).* Screening invitees were mailed the standard invitation 2 weeks before their screening kit. The invitation was sent in an NHS envelope and contained an invitation letter and ‘The Facts’ booklet. After 2 weeks, invitees were mailed a gFOBt kit with a standard instructional leaflet.

*Intervention group: Standard Information booklet + Gist leaflet (SI + Gist)* ***.*** People in the intervention group received the Gist leaflet 2 weeks before the screening kit in the same envelope as the standard booklet. A copy of the Gist leaflet can be found in Additional file 1: Fig. S1. The Gist leaflet was developed using the General Medical Council guidelines [18]. The development process is described elsewhere [17, 19]. Structured interviews identified areas of the standard information booklet susceptible to being misunderstood [20]. We addressed problematic areas in the Gist leaflet by using principles of information design [16]. The Gist leaflet underwent user-testing to refine its readability and comprehensibility [17]. The acceptability of the Gist leaflet and its effect on knowledge was demonstrated in a randomised controlled trial (*n* = 964) with adults from deprived areas [19]. The organisation and schedule of the trial is shown in Additional file 2: Fig. S2.

### Randomisation

Randomisation was by day of invitation, with ‘day within Hub’ constituting the randomisation unit (hub-day). Randomisation occurred over 10 consecutive days in November 2012. Two weeks prior, the randomisation sequence was generated for each hub-day by the trial statistician and sent to the organisations handling the mailing: Real Digital International (RDI) for the Southern, London and Eastern hubs, and an ‘in house’ system for the North-East and North West Hubs. For each hub, ten random numbers were generated. Hub-days above the median random number were allocated to intervention and hub days below to control. Blinding of hubs was not possible, but bias was unlikely due to the lack of contact with subjects [21]. Invited subjects were unaware of a comparator condition unless a member of their household was also invited during the study period.

### Outcome measures

Screening uptake was defined as the return of a gFOBt kit within 18 weeks of the invitation that led to a ‘definitive’ test result of either ‘normal’ (i.e. no further investigation required) or ‘abnormal’ (i.e. referral for further testing, usually colonoscopy) by the date of data extraction (18 weeks after the last day of the intervention).

People were classified as not adequately screened if their first result was ‘unclear’, ‘spoilt’, or a technical ‘failure’, and they did not complete a subsequent kit. Screening uptake was therefore computed using data on the outcomes of all screening kits completed, and the denominator was the number of invited subjects. We compared the effectiveness of the ‘SI + Gist’ condition against ‘SI’ alone. The primary outcome was the gradient in uptake rates over quintiles of SES. Secondary outcomes were (i) overall uptake; (ii) SES differences in uptake between the study groups within age, gender, hub and screening round sub-groups; (iii) time taken to return gFOBt; (iv) proportion of spoilt kits; (v) screening result; and (vi) diagnostic outcome for those with abnormal gFOBt results.

We used the Index of Multiple Deprivation (IMD) 2010 associated with each individual’s home address to classify SES [22]. IMD is an area-based measure that combines seven domains (e.g. income, employment, education) into a single deprivation score. IMD scores were grouped into quintiles from 1 (least deprived) to 5 (most deprived). Data were available on age at invitation, gender, hub, and screening round. The latter was categorised as incident screening (individual had previously participated) and prevalent screening (individual had not previously participated). The prevalent round of screening was further divided into those who had not previously been invited to screening (first time invitees) and those who had previously declined screening (previous non-responders).

### Statistical considerations

The target sample size was based on achieving a reduction in the SES gradient associated with screening uptake. We assumed a fixed proportional effect in each hub and estimated an average increase of 3 percentage points, based on increasing uptake by 5 percentage points in the lowest (fifth) IMD quintile (low SES) and 1 percentage point in the highest (first) quintile (high SES), giving an overall 1–2–3-4-5 percentage point difference by quintile [23]. This is considered feasible screening uptake research [24].

A published power calculation is available elsewhere [25]. Briefly, with 90% power and 5% statistical significance, 46,000 individuals (23,000 per arm) were required to detect a 1–2–3-4-5 percentage point difference in uptake in the least to most deprived IMD quintile, respectively. However, due to the volume of invitations sent out by each hub per week (70,000–80,000), this sample would be achieved within 5 days. This number of clusters would have a risk of bias [26]. The intervention therefore ran for 10 days, providing a sample of 140,000–165,000.

The primary outcome was analysed by logistic regression in a univariable model, and then a multivariable model adjusting for age, gender, hub and screening round. *P*-values and 95% confidence intervals (CIs) were calculated using conservative variance estimation to allow for potential clustering effects in randomisation [21, 26]. The association between the proportion of people adequately screened and SES was assessed by including an interaction term for trial arm and IMD score (as a continuous variable) in the models. The association was also investigated by stratifying according to age at invite, gender, hub and screening round. Analysis was performed on an intention-to-treat basis using SAS v9.3 (SAS Institute Inc., Cary, NC, USA) and Stata v12.1 (StataCorp LP, College Station, TX, USA).

### Availability of data and materials

The study data are available to the corresponding author (CvW) and are not available for release as they contain patient-identifiable information.

### Assessment of concurrent initiatives

To determine whether the intervention was affected by other initiatives, we surveyed national and local research and health promotion activities during the trial. We surveyed key informants, including Quality Assurance Reference Centre (QARC) Directors, a National Awareness and Early Diagnosis Initiative (NAEDI) representative, Specialist Screening Practitioners (SSPs), BCSP Programme Managers, the National Cancer Research Network and Strategic Clinical Network representatives.

## Results

**Fig. 1:**
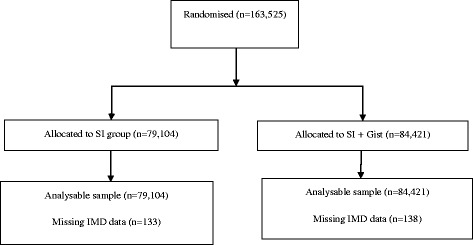
Flow of participants through the trial

Between the 5th and 16th of November 2012, 163,525 individuals were allocated to either the ‘SI’ intervention (*n* = 79,104) or the ‘SI + Gist’ group (*n* = 84,421) based on the hub-day (Fig. 1). Baseline characteristics were similar in the groups (Table 1). Over half of all invitees (*n* = 57.4%) were defined as adequately screened. Median (range) time to return the kit was 22 days (11–142) for the SI group and 23 days (12–142) for the SI + Gist group. The proportion of spoilt test kits (*n* = 1256, 0.8%) or undelivered mail (*n* = 822, 0.5%) was small and similar across trial arms and IMD quintiles.

**Table 1:** Baseline characteristics

Variables	SI + Gist *N* = 84,421	SI *N* = 79,104
	median (range)	median (range)
Age at invite (in years)	66.0 (59.0–74.0)	66.0 (59.0–74.0)
IMD deprivation score	14.9 (0.5–87.8)	14.8 (0.5–87.8)
	% (n)	% (n)
Gender		
Female	51.2 (43195)	51.4 (40671)
Male	48.8 (41226)	48.6 (38433)
Socioeconomic status quintile		
Least deprived (0–8.49)	22.6 (19055)	23.5 (18554)
2nd quintile (8.50–13.79)	23.5 (19787)	23.2 (18295)
3rd quintile (13.80–21.35)	21.7 (18320)	20.3 (15993)
4th quintile (21.36–34.17)	17.5 (14747)	17.1 (13469)
Most deprived (34.18–87.80)	14.7 (12374)	16.0 (12660)
*Missing*	*138*	*133*
Hub		
Midlands & North West	26.6 (22469)	30.8 (24369)
Southern	24.5 (20651)	26.6 (21004)
London	8.8 (7416)	8.4 (6636)
North East	16.1 (13614)	16.3 (12858)
Eastern	24.0 (20271)	18.0 (14237)
Screening round		
Incident episodes	53.3 (45019)	53.3 (42143)
Prevalent first time invitees	15.4 (13034)	15.7 (12410)
Prevalent previous non-responders	31.2 (26368)	31.0 (24551)

The proportion of adequately screened individuals increased by 0.38 percentage points overall in the Gist condition: SI + Gist = 57.6% versus SI = 57.3% (OR = 1.02, 95% CI: 0.92–1.13, *p* = 0.77). The proportion screened decreased as deprivation score increased in both arms (SI + Gist: 65.8% to 43.0% and SI: 65.6% to 42.0%), but was similar between the trial groups in each IMD quintile, providing no evidence that the intervention reduced inequalities (interaction *p*-value = 0.48) (Table 2).

**Table 2:** Proportion of individuals who were adequately screened^a^, according to socioeconomic status quintile^b^

Variable	SI + Gist* *N* = 84,421	SI* *N* = 79,104
	% (n)	% (n)
Adequately screened:	57.6 (48653)	57.3 (45290)
1st quintile (least deprived)	65.8 (12547)	65.6 (12178)
2nd quintile	62.2 (12305)	62.4 (11412)
3rd quintile	58.6 (10732)	58.4 (9335)
4th quintile	52.0 (7663)	51.9 (6987)
5th quintile (most deprived)	43.0 (5322)	42.0 (5316)

^a^ Returned a gFOBt kit within 18 weeks of the invitation that led to a ‘definitive’ test result of either ‘normal’ (i.e. no further investigation required) or ‘abnormal’ (i.e. requiring referral for further testing, usually colonoscopy) by the date of data extraction (18 weeks after the last day of the intervention)

^b^ 271 (138 SI + Gist and 133 SI) individuals missing socioeconomic status, 146 of these were adequately screened (84 SI + Gist and 62 SI)

*Comparison between trials groups: OR = 1.02, 95% CI: 0.92–1.13, *p* = 0.77

*Comparison between trials groups adjusting for age, gender, hub and screening round: OR = 1.03, 95% CI: 0.99–1.06, *p* = 0.15

There was no difference in the proportion of individuals adequately screened between the trial groups by age at invitation (<65 years OR = 1.03, 95% CI: 0.94–1.13, *p* = 0.52; 65–69 years OR = 0.98, 95% CI: 0.85–1.13, *p* = 0.83; 70+ years OR = 1.04, 95% CI: 0.90–1.19, *p* = 0.64). The proportion screened was generally lower in younger individuals (<65 years 54.6% vs. 65–69 years 60.9% vs. 70+ years 57.3%), and decreased with deprivation in both arms (Table 3). There was no evidence of an association between the trial arm and deprivation score on the proportion screened in any age group (interaction *p*-value: <65 years *p* = 0.86; 65–69 years *p* = 0.47; 70+ years *p* = 0.46).

**Table 3:** Proportion of individuals who were adequately screened^a^, according to socioeconomic status quintile^b^ and median age at invite

	Age at invite <65 years*		Age at invite 65–69 years*		Age at invite 70+ years*	
	SI + Gist *N* = 35,920	SI *N* = 33,589	SI + Gist *N* = 30,707	SI *N* = 28,379	SI + Gist *N* = 17,794	SI *N* = 17,136
	% (n)	% (n)	% (n)	% (n)	% (n)	% (n)
Adequately screened:	54.9 (19727)	54.2 (18200)	60.8 (18657)	61.1 (17346)	57.7 (10269)	56.9 (9744)
1st quintile (least deprived)	63.6 (5135)	62.9 (4883)	69.1 (4740)	69.0 (4655)	64.9 (2672)	65.3 (2640)
2nd quintile	59.4 (4924)	59.0 (4449)	64.8 (4751)	66.5 (4476)	63.1 (2630)	61.9 (2487)
3rd quintile	54.9 (4201)	55.3 (3762)	61.8 (4224)	61.7 (3488)	60.1 (2307)	59.0 (2085)
4th quintile	50.0 (3206)	48.7 (2880)	55.3 (2961)	56.7 (2706)	50.3 (1496)	50.3 (1401)
5th quintile (most deprived)	41.0 (2226)	39.9 (2199)	45.5 (1946)	45.1 (1996)	43.1 (1150)	41.1 (1121)

^a^Returned a gFOBt kit within 18 weeks of the invitation that led to a ‘definitive’ test result of either ‘normal’ (i.e. no further investigation required) or ‘abnormal’ (i.e. requiring referral for further testing, usually colonoscopy) by the date of data extraction (18 weeks after the last day of the intervention)

^b^271 (138 intervention and 133 control) individuals missing socioeconomic status

*Comparison between trials groups within age at invite group: <65 years (OR = 1.03, 95% CI: 0.94–1.13, *p* = 0.52); 65–69 years (OR = 0.98, 95% CI: 0.85–1.13, *p* = 0.83); 70+ years (OR = 1.04, 95% CI: 0.90–1.19, *p* = 0.64)

*Comparison between trials groups within age at invite group adjusting for gender, hub and screening round: <65 years (OR = 1.03, 95% CI: 0.99–1.07, *p* = 0.13); 65–69 years (OR = 1.00, 95% CI: 0.93–1.07, *p* = 0.93); 70+ years (OR = 1.06, 95% CI: 0.99–1.13, *p* = 0.08)

There was little difference in the overall proportion adequately screened between the trial arms by gender (men OR = 1.02, 95% CI: 0.92–1.14, *p* = 0.65; women OR = 1.01, 95% CI: 0.91–1.12, *p* = 0.89). The proportion screened was lower in men than women (55.7% vs. 59.1) and decreased with deprivation in both arms (Table 4), but with no arm by deprivation interaction for men (*p* = 0.33) or women (*p* = 0.78).

**Table 4:** Proportion of individuals who were adequately screened^a^, according to socioeconomic status quintile^b^ and gender

	Males*		Females*	
	SI + Gist *N* = 41,226	SI *N* = 38,433	SI + Gist *N* = 43,195	SI *N* = 40,671
	% (n)	% (n)	% (n)	% (n)
Adequately screened:	56.0 (23068)	55.4 (21273)	59.2 (25585)	59.1 (24017)
1st quintile (least deprived)	64.1 (5917)	64.3 (5762)	67.4 (6630)	66.9 (6416)
2nd quintile	60.6 (5863)	59.9 (5287)	63.7 (6442)	64.7 (6125)
3rd quintile	56.5 (5050)	56.5 (4385)	60.5 (5682)	60.1 (4950)
4th quintile	50.3 (3602)	49.8 (3274)	53.5 (4061)	53.8 (3713)
5th quintile (most deprived)	42.1 (2602)	40.6 (2535)	43.9 (2720)	43.4 (2781)

^a^Returned a gFOBt kit within 18 weeks of the invitation that led to a ‘definitive’ test result of either ‘normal’ (i.e. no further investigation required) or ‘abnormal’ (i.e. requiring referral for further testing, usually colonoscopy) by the date of data extraction (18 weeks after the last day of the intervention)

^b^ 271 (138 SI + Gist and 133 SI) individuals missing socioeconomic status, 146 of these were adequately screened (84 SI + Gist and 62 SI)

*Comparison between trials groups within each gender: Males (OR = 1.02, 95% CI: 0.92–1.14, *p* = 0.65); Females (OR = 1.01, 95% CI: 0.91–1.12, *p* = 0.89)

*Comparison between trials groups within each gender adjusting for age, hub and screening round: Males (OR = 1.05, 95% CI: 1.01–1.10, *p* = 0.03); Females (OR = 1.00, 95% CI: 0.96–1.05, *p* = 0.91)

The proportion adequately screened was lower in people who had not previously taken part in CRC screening (prevalent first time invitees OR = 1.06, 95% CI: 0.96–1.16, *p* = 0.23; prevalent previous non-responders OR = 1.03, 95% CI: 0.94–1.13, *p* = 0.50; incident episodes OR = 1.01, 95% CI: 0.95–1.08, *p* = 0.67), and decreased with deprivation in both arms (Table 5). There was no difference in the overall proportion screened between the trial arms according to previous participation, nor an interaction with deprivation score (interaction *p*-value: prevalent first time invitees *p* = 0.13; prevalent previous non-responders *p* = 0.09; incident episodes *p* = 0.38).

**Table 5:** Proportion of individuals who were adequately screened^a^, according to socioeconomic status quintile^b^ and screening round

	Prevalent first time invitees*		Prevalent previous non-responders*		Incident episodes*	
	SI + Gist *N* = 13,034	SI *N* = 12,410	SI + Gist *N* = 26,368	SI *N* = 24,551	SI + Gist *N* = 45,019	SI *N* = 42,143
	% (n)	% (n)	% (n)	% (n)	% (n)	% (n)
Adequately screened:	49.6 (6466)	48.2 (5981)	14.5 (3836)	14.2 (3479)	85.2 (38351)	85.0 (35830)
1st quintile (least deprived)	58.3 (1708)	56.1 (1541)	16.9 (792)	17.8 (796)	87.9 (10047)	86.9 (9841)
2nd quintile	55.6 (1610)	53.4 (1473)	16.2 (898)	15.9 (790)	86.3 (9797)	86.6 (9149)
3rd quintile	49.7 (1352)	49.4 (1270)	15.5 (874)	15.3 (741)	85.4 (8506)	85.4 (7324)
4th quintile	43.7 (995)	42.3 (943)	13.0 (683)	12.7 (596)	83.1 (5985)	83.4 (5448)
5th quintile (most deprived)	36.0 (786)	35.9 (746)	11.2 (580)	10.0 (549)	79.2 (3956)	79.1 (4021)

^a^Returned a gFOBt kit within 18 weeks of the invitation that led to a ‘definitive’ test result of either ‘normal’ (i.e. no further investigation required) or ‘abnormal’ (i.e. requiring referral for further testing, usually colonoscopy) by the date of data extraction (18 weeks after the last day of the intervention)

^b^271 (138 intervention and 133 control) individuals missing socioeconomic status

*Comparison between trials groups within each screening round: Prevalent first time invitees (OR = 1.06, 95% CI: 0.96–1.16, *p* = 0.23); Prevalent previous non-responders (OR = 1.03, 95% CI: 0.94–1.13, *p* = 0.50); Incident episodes (OR = 1.01, 95% CI: 0.95–1.08, *p* = 0.67)

*Comparison between trials groups within each screening round adjusting for age, gender and hub: Prevalent first time invitees (OR = 1.04, 95% CI: 0.98–1.10, *p* = 0.17); Prevalent previous non-responders (OR = 1.03, 95% CI: 0.96–1.09, *p* = 0.44); Incident episodes (OR = 1.01, 95% CI: 0.96–1.07, *p* = 0.73)

In the Southern Hub, overall uptake was lower in the SI + Gist group (OR = 0.89, 95% CI: 0.84–0.94, *p* < 0.01) and in each deprivation quintile, although there was no effect after adjusting for baseline characteristics (OR = 1.01, 95% CI: 0.95–1.07, *p* = 0.75). There was no difference in uptake between trial arms in the other hubs (Midlands & North West OR = 1.01, 95% CI: 0.83–1.24, *p* = 0.89; London OR = 0.99, 95% CI: 0.64–1.52, *p* = 0.96; North East OR = 1.03, 95% CI: 0.89–1.19, *p* = 0.68; Eastern OR = 1.18, 95% CI: 0.97–1.43, *p* = 0.09). An interaction with deprivation score was seen in the London Hub (*p* < 0.01), but the proportion screened was lower in the SI + Gist group than the SI group among the most deprived individuals and the reverse was seen in the least deprived group. This was non-significant after adjusting for baseline characteristics (*p* = 0.82). There was no interaction with deprivation score in the Midlands & North West (*p* = 0.10), Southern (*p* = 0.93), North East (*p* = 0.09), or Eastern (*p* = 0.58) hubs (Table 6).

**Table 6:** Proportion of individuals who were adequately screened^a^, according to socioeconomic status quintile^b^ and hub

	Midlands & North West*		Southern*		London*		North East*		Eastern*	
	SI + Gist *N* = 22,469	SI *N* = 24,369	SI + Gist *N* = 20,651	SI *N* = 21,004	SI + Gist *N* = 7416	SI *N* = 6636	SI + Gist *N* = 13,614	SI *N* = 12,858	SI + Gist *N* = 20,271	SI *N* = 14,237
	% (n)	% (n)	% (n)	% (n)	% (n)	% (n)	% (n)	% (n)	% (n)	% (n)
Adequately screened:	54.9 (12336)	54.6 (13297)	59.0 (12177)	61.9 (12991)	55.0 (4078)	55.2 (3665)	58.2 (7918)	57.4 (7382)	59.9 (12144)	55.9 (7955)
1st quintile (least deprived)	63.9 (2645)	65.9 (2852)	65.7 (4001)	67.1 (4884)	71.6 (752)	66.4 (558)	67.8 (1588)	67.7 (1422)	65.5 (3561)	61.4 (2462)
2nd quintile	61.4 (3037)	62.3 (3194)	60.5 (3170)	63.4 (3395)	62.0 (876)	60.5 (764)	65.1 (1831)	65.6 (1782)	63.2 (3391)	59.5 (2277)
3rd quintile	56.7 (2607)	57.6 (2693)	57.7 (2807)	60.2 (2760)	56.5 (907)	56.3 (783)	62.4 (1673)	62.2 (1533)	59.8 (2738)	54.4 (1566)
4th quintile	50.7 (1953)	50.5 (2136)	51.4 (1602)	53.5 (1433)	51.7 (983)	53.1 (950)	53.3 (1423)	53.2 (1353)	53.0 (1702)	50.0 (1115)
5th quintile (most deprived)	42.3 (2077)	40.3 (2403)	44.2 (569)	46.5 (495)	38.7 (555)	45.3 (610)	45.0 (1393)	42.5 (1283)	44.1 (728)	41.5 (525)

^a^ Returned a gFOBt kit within 18 weeks of the invitation that led to a ‘definitive’ test result of either ‘normal’ (i.e. no further investigation required) or ‘abnormal’ (i.e. requiring referral for further testing, usually colonoscopy) by the date of data extraction (18 weeks after the last day of the intervention).^b^ 271 (138 SI + Gist and 133 SI) individuals missing socioeconomic status, 146 of these were adequately screened (84 SI + Gist and 62 SI)

*Comparison between trials groups within each hub: Midlands & North West (OR = 1.01, 95% CI: 0.83–1.24, *p* = 0.89); Southern (OR = 0.89, 95% CI: 0.84–0.94, *p* < 0.01); London (OR = 0.99, 95% CI: 0.64–1.52, *p* = 0.96); North East (OR = 1.03, 95% CI: 0.89–1.19, *p* = 0.68); Eastern (OR = 1.18, 95% CI: 0.97–1.43, *p* = 0.09)

*Comparison between trials groups within each hub adjusting for age, gender and screening round: Midlands & North West (OR = 1.01, 95% CI: 0.93–1.09, *p* = 0.85); Southern (OR = 1.01, 95% CI: 0.95–1.07, *p* = 0.75); London (OR = 1.01, 95% CI: 0.88–1.16, *p* = 0.93); North East (OR = 1.08, 95% CI: 0.99–1.17, *p* = 0.09); Eastern (OR = 1.03, 95% CI: 0.96–1.10, *p* = 0.40)

Of the 93,943 individuals adequately screened, 1703 (1.8%) had a definitive abnormal result. Diagnostic outcomes are known for 1377 (80.9%) individuals with an abnormal screening result (Additional file 3: Table S1). We received details of 62 health promotion activities and 17 research projects being undertaken during this trial. These initiatives were not limited to occurring on the same days the Gist leaflet was sent out.

## Discussion

In this randomised controlled trial embedded within the English NHS BCSP, a supplementary Gist leaflet containing essential and simple information about CRC screening combined with the standard information booklet did not reduce SES inequalities in uptake compared with the existing materials alone. The Gist leaflet did not affect overall uptake and there were no differences in the SES gradient between the study groups within age, gender, screening status or hub sub-groups. Screening uptake was lower in the intervention arm of the Southern hub, which generally serves more affluent areas. This effect was removed in controlled analyses suggesting the individual characteristics of each hub may not support a ‘one size fits all’ approach. The intervention was not affected by concurrent initiatives.

Several studies have shown that people, particularly older adults, have a preference for extracting gist-like representations from health information, but this is among the first attempts to evaluate information materials guided by Fuzzy-Trace Theory [27, 28]. Several attempts have been made to increase screening uptake using mailed materials informed by a range of psychological theories, and these have resulted in positive, [29–31] negative [32] and null outcomes [33–35]. To our knowledge, no study has specifically attempted to reduce the socioeconomic gradient in screening uptake. This study was part of a programme of work evaluating three other invitation strategies, a general practice endorsement, an enhanced reminder and a narrative leaflet describing people’s stories about screening [25]. Only the enhanced reminder affected the SES gradient in uptake and marginal gains were observed in overall uptake when using a general practice endorsement [36]. Despite the strong theoretical backgrounds and extensive pre-testing of these interventions, the design of effective strategies to promote colorectal screening uptake is challenging.

Among the strengths of our trial were its national coverage, substantial power to detect small differences in uptake, and an intervention, which, if effective, could easily and cost-effectively be incorporated into the existing BCSP. We used novel, innovative methods to develop the Gist leaflet, and carried out extensive user testing and piloting to demonstrate its accessibility to adults with basic literacy skills [17, 19]. A major limitation was that we had to deliver the Gist leaflet as a supplement to, rather than a replacement for, the existing leaflet. Given that complex written information is challenging for individuals with limited literacy [37, 38], the effect of the Gist leaflet may have been undermined by the increase in the volume of material sent. Our findings should therefore not preclude future studies evaluating the impact of a standalone gist leaflet. We were also unable to record knowledge and attitudes, and therefore the extent to which informed decision-making was affected is unknown. Furthermore, we did not attempt to address broader attitudes towards cancer, such as cancer fatalism, which are known to affect participation [6]. While low literacy may be an important barrier to colorectal screening participation, it is possible that other factors not addressed by the gist leaflet may be more influential.

## Conclusions

In conclusion, despite an extensive testing process our supplementary information leaflet, giving the ‘gist’ of the NHS BCSP in England neither increased overall uptake nor reduced socioeconomic inequalities in screening. Alternative strategies may be required to ensure groups from lower socioeconomic status backgrounds, including those with low levels of literacy, participate at similar rates to their more affluent counterparts. The effectiveness of the Gist leaflet when used alone is unknown and should be investigated.

## Additional files

Additional file 1: Figure S1. ‘Gist’ leaflet. (DOCX 184 kb)

Additional file 2: Figure S2. Organisation and schedule of the national trial. (PDF 107 kb)

Additional file 3: Diagnostic outcome for adequately screened† individuals with a definitive abnormal result*. (DOCX 16 kb)

## Abbreviations

CRC: Colorectal cancer
gFOBt: Guaiac-based Faecal Occult Blood test
GP: General Practitioner
IMD: Index of Multiple Deprivation
NAEDI: National Awareness and Early Diagnosis Initiative
NHS BCSP: National Health Service Bowel Cancer Screening Programme
NIGB: National Information Governance Board
QARC: Quality Assurance Reference Centre
REC: Research ethics committee
SES: Socioeconomic Status
SI + Gist: Standard information booklet and the Gist leaflet
SI: Standard information booklet
