# Supplementary figures and images for: Reducing the socioeconomic gradient in uptake of the NHS bowel cancer screening Programme using a simplified supplementary information leaflet: a cluster-randomised trial
Source: BMC Cancer. 2017 Aug 14;17:543. doi: 10.1186/s12885-017-3512-1 (PMC5556676; doi:10.1186/s12885-017-3512-1)

Supplementary Figure 1: ‘Gist Leaflet’


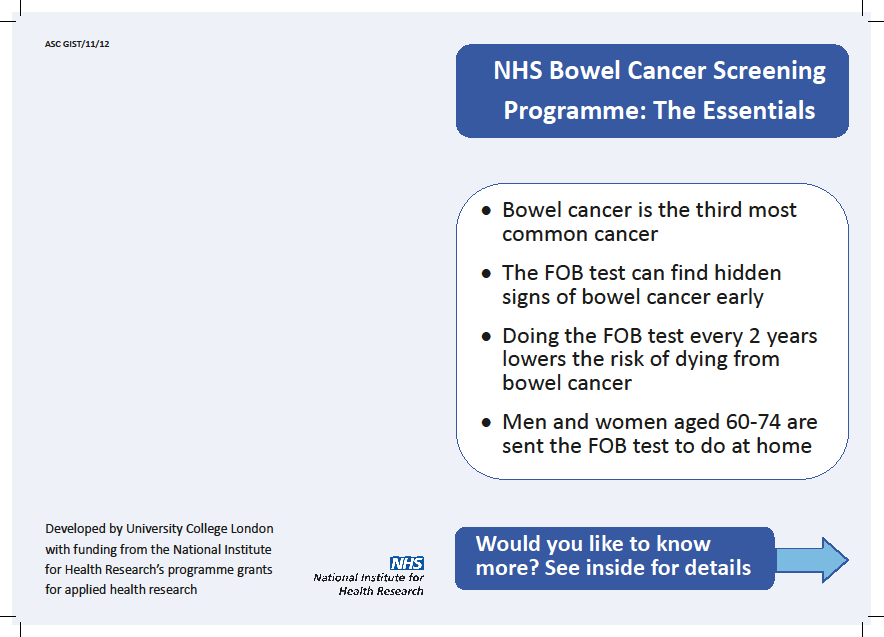


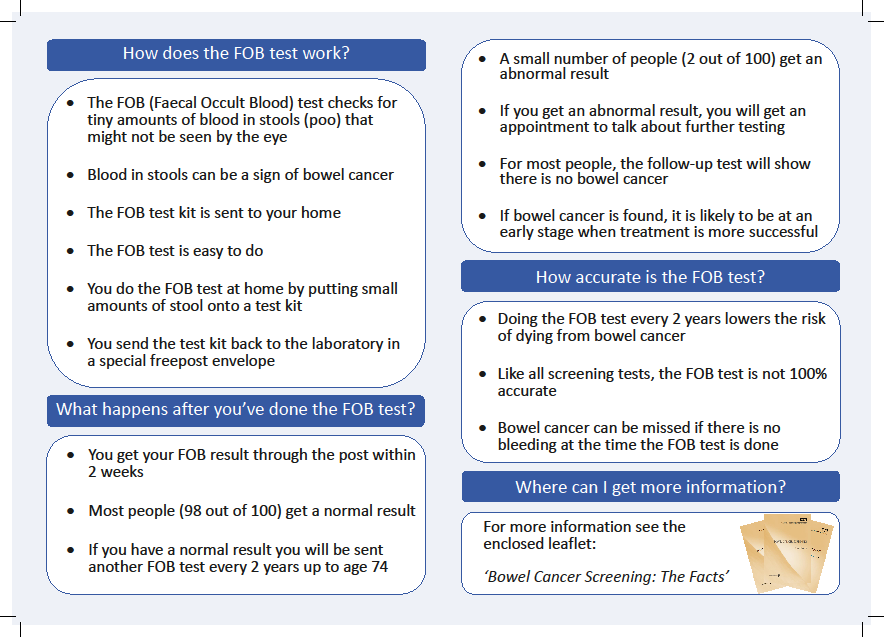

Supplement: Supplementary file 1 — ‘Gist’ leaflet. (DOCX 184 kb) [file 12885_2017_3512_MOESM1_ESM.docx]

Supplementary Figure 2

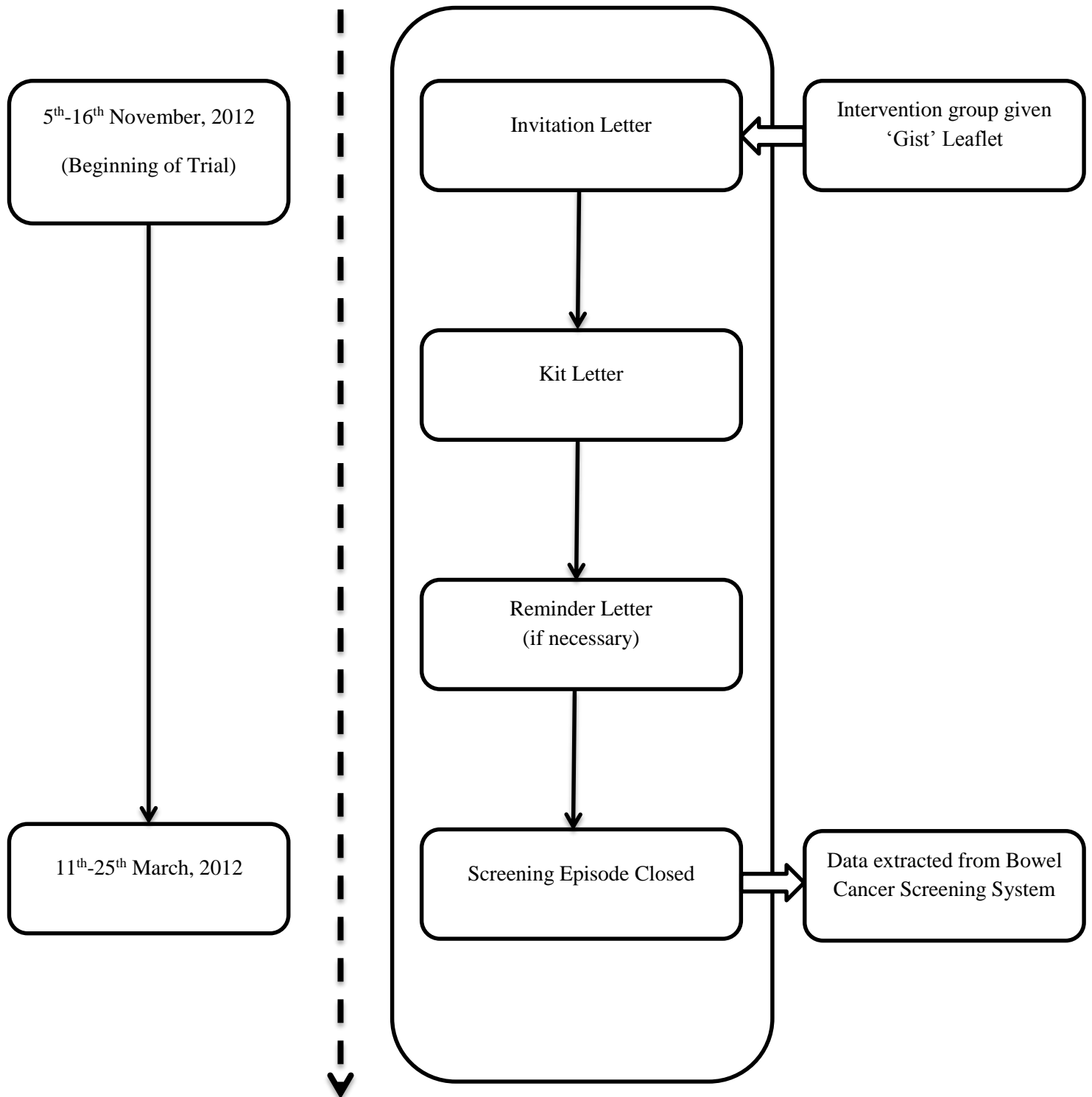

Supplement: Supplementary file 2 — Organisation and schedule of the national trial. (PDF 107 kb) [file 12885_2017_3512_MOESM2_ESM.pdf]
