# Supplementary material for: Reducing the socioeconomic gradient in uptake of the NHS bowel cancer screening Programme using a simplified supplementary information leaflet: a cluster-randomised trial
Source: BMC Cancer. 2017 Aug 14;17:543. doi: 10.1186/s12885-017-3512-1 (PMC5556676; doi:10.1186/s12885-017-3512-1)
Supplement: Supplementary file 3 — Diagnostic outcome for adequately screened† individuals with a definitive abnormal result*. (DOCX 16 kb) [file 12885_2017_3512_MOESM3_ESM.docx]

Supplementary Table 1. Diagnostic outcome for adequately screened† individuals with a definitive abnormal result*

| **Variable** | **SI + Gist**  **N=911** | **SI**  **N=792** |
| --- | --- | --- |
|  |  |  |
|  | **% (n)** | **% (n)** |
| **Diagnostic outcome known:** | **81.7 (744)** | **79.9 (633)** |
| Abnormal | 25.9 (236) | 24.2 (192) |
| Cancer detected | 5.2 (47) | 6.6 (52) |
| High-risk adenoma | 8.2 (75) | 7.1 (56) |
| Intermediate-risk adenoma | 12.8 (117) | 11.1 (88) |
| Low-risk adenoma | 15.5 (141) | 15.8 (125) |
| Normal (no abnormalities found) | 14.1 (128) | 15.2 (120) |
|  |  |  |
| **Diagnostic outcome unknown** | **18.3 (167)** | **20.1 (159)** |
| Did not attend SSP appointment | 4.8 (44) | 6.6 (52) |
| Did not book colonoscopy or other test | 7.6 (69) | 8.1 (64) |
| Did not attend colonoscopy or other test | 4.2 (38) | 4.2 (33) |
| No result | 1.8 (16) | 1.3 (10) |
|  |  |  |

† Returned a gFOBt kit within 18 weeks of the invitation that led to a ‘definitive’ test result of either ‘normal’ (i.e. no further investigation required) or ‘abnormal’ (i.e. requiring referral for further testing, usually colonoscopy) by the date of data extraction (18 weeks after the last day of the intervention).‡ 271 (138 SI+Gist and 133 SI) individuals missing socioeconomic status, 146 of these were adequately screened (84 SI+Gist and 62 SI)

*Some individuals will have reached an outcome after the date of data extraction.
